# A noninvasive strategy for multi-disease diagnosis via multi-sensor platform: integrative analysis of five years of exhaled breath–based diagnostics for seven diseases

**DOI:** 10.3389/fbioe.2025.1750962

**Published:** 2026-01-06

**Authors:** Alessandro Zompanti, Giorgio Pennazza, Simone Grasso, Anna Sabatini, Maria Vittoria Di Loreto, Costanza Cenerini, Ludovica La Monica, Luca Vollero, Raffaele Antonelli Incalzi, Claudio Pedone, Panaiotis Finamore, Simone Scarlata, Antonio De Vincentis, Antonio Picardi, Pierfilippo Crucitti, Filippo Longo, Gaetano Rocco, Andrea Segreti, Francesco Grigioni, Marco Santonico

**Affiliations:** 1 Unit of Electronics for Sensor Systems, Department of Engineering, Università Campus Bio-Medico di Roma, Rome, Italy; 2 Unit of Electronics for Sensor Systems, Department of Science and Technology for Sustainable Development and One-Health, Università Campus Bio-Medico di Roma, Rome, Italy; 3 Unit of Computational Systems and Bioinformatics, Department of Engineering, Università Campus Bio-Medico di Roma, Rome, Italy; 4 Geriatrics, Fondazione Policlinico Universitario Campus Bio-Medico, Roma, Italy; 5 Research Unit of Internal Medicine, Department of Medicine and Surgery, Università Campus Bio-Medico di Roma, Roma, Italy; 6 Department of Clinical Medicine and Hepatology, Università Campus Bio-Medico di Roma, Rome, Italy; 7 Department of Thoracic Surgery, Fondazione Policlinico Universitario Campus Bio-Medico di Roma, Rome, Italy; 8 Thoracic Service, Department of Surgery, Memorial Sloan Kettering Cancer Center, New York, NY, United States; 9 Department of Cardiovascular Sciences, Fondazione Policlinico Universitario Campus Bio-Medico di Roma, Rome, Italy; 10 Department of Movement, Human and Health Sciences, University of Rome “Foro Italico”, Rome, Italy

**Keywords:** exhaled breath, multivariate analysis, non invasive assessment, quart crystal microbalance, sensor array

## Abstract

Sensors used for collecting high-value physiological and biochemical data strongly support a precision medicine approach, by enabling the integration of these complex datasets with routine clinical outcomes to provide more accurate diagnostic and prognostic evaluations. Building on extensive experience in this field, the authors have developed multi-sensor technologies designed to analyze multiple biological fluids, with a particular focus on exhaled breath both in as it is and processed in liquid media. These technologies have been implemented in a large-scale clinical study involving 863 patients affected by seven different diseases, allowing for the acquisition of heterogeneous data suitable for computational modeling and the identification of disease-related characteristics. By integrating multiple sensors and analyzing diverse breath samples, this work aims to generate a comprehensive reference library of breathprints and thereby advance the clinical applicability of breath analysis. The study demonstrates the potential of this multi-omic, multisensory approach to differentiate healthy individuals from patients with various respiratory, cardiovascular, and metabolic disorders, while pioneering investigations of exhaled breath in liquid media—although conducted on a smaller patient cohort—highlight promising opportunities for technological innovation in multisensory diagnostics. While the overall results support the feasibility and potential impact of this methodology, further research will be required to refine the technique, enlarge patient cohorts, and improve the accuracy and specificity of disease detection.

## 1 Introduction

A Google search carried out on the term '-omics' in October 2024 yielded 829 items. The numerous entries were most diverse, but the most recurrent ones referred to the biomedical field. In the field of biomedical research, the utilization of the term ‘omics’ has become a semantic shortcut to introduce a new method/instrument in the frame of the innovative diagnostic technologies, obtaining a tremendous heterogeneity. According to Omenn et al. (2012) “Omics is a term encompassing multiple molecular disciplines that involve the characterization of global sets of biological molecules such as DNAs, RNAs”, but in practice this term is often generalized to every kind of health-related data, with the aim of characterize patients, populations and diseases by mean of a fingerprint/handprint. Handprint is here intended as the result of an integrated approach on biological systems by combining medical records from heterogeneous sources to identify classes of patients with a specific disease through potentially increasing phenotyping accuracy (Goldman, 2012). It is useful to better clarify the background of the different (technological/instrumental) source of data, from generic health records (pertaining to routine clinical exams or to experimental methods) to the scientific evidence with a clear biological rationale (Wu et al., 2016). Google based definition is here used as a preliminary reference point to illustrate the broader confusion that exists in public discourse, which underscores the need for a proper scientific interpretation. From a practical standpoint, all clinical laboratory tests based on a single analyte lacking a clear biological rationale are considered non-omics. However, all such techniques could be unified in the wholeness of the -omics suffix by the presence of a common goal (the ‘forward ground’) regardless of a well-marked difference on the starting point (the background).

O*mics* disciplines (such as genomics, proteomics, metabolomics, and transcriptomics) can contribute with a foundational knowledge paving the way for a new paradigm of health-state monitoring that supports the implementation of precision medicine.

In the landscape of precision medicine, sensors occupy a central and strategic role. This is clearly demonstrated by keyword clustering analyses (e.g., as shown in Silvera-Tawil et al. (2020)), where the term “sensor” appears among the largest and most interconnected nodes. Such findings underscore the foundational role of sensors in biomedical technologies.

Sensors act as the primary interface between biological phenomena and digital data. Any physiological signal or molecular presence—whether DNA fragments (genomics), protein markers (proteomics), metabolic byproducts (metabolomics), or RNA sequences (transcriptomics)—must be captured and translated into measurable electrical signals. This process is made possible through sensing blocks and transduction mechanisms, which vary according to the application but rely on well-established physical principles.

Given the diverse range of biological inputs from different *omics* layers, the continued evolution of sensor technology is imperative. Key innovation priorities include enhanced electronic interfaces, with the development of sensors with higher resolution, sensitivity, and reproducibility to capture subtle biological variations with clinical relevance; energy efficiency and sustainability, with the integration of low-power electronics and energy-harvesting techniques to enable long-term monitoring with minimal environmental and operational costs; robust connectivity and integration, establishing reliable network interfaces that ensure seamless data transmission within scalable, sustainable production frameworks.

Furthermore, AI (Artificial Intelligence) tools can help interpret the complex datasets generated by *omics*-driven sensors, to allow the identification of underlying biological mechanisms.

In the last 10 years, the authors have followed an innovative path characterized by ‘multidimensionality’: multi-sensors technologies (different working principles for measuring liquids and volatiles) (Santonico et al., 2013; Pennazza et al., 2018a; Pennazza et al., 2018b); multi-specimen (different biological fluids: exhaled breath, urine, saliva …) (Pipita et al., 2020; Pennazza et al., 2014; Schirinzi et al., 2021; Lelli et al., 2018); multi-diseases (many different diseases and their comorbidities) (Rocco et al., 2016; Scarlata et al., 2017; Finamore et al., 2018a). This strategy could be defined as multi-omics, which is the approach followed by many international networks and projects to obtain a handprint starting from different omics (Ahmed et al., 2018). We have pursued our multi-omics strategy through a series of technological steps: 1) The sensor system, called BIONOTE (Santonico et al., 2013), is multi-sensor by design: Quartz Crystal Microbalances for volatiles, Cyclic voltammetry for Liquids, LED-Photodetector for optical detection; 2) An experiment of multi-acquisition and analysis have been already addressed to microbioma (De Vincentis et al., 2021); 3) A multi-disease clinical study based on a multi-sensor approach consisting in the characterization of exhaled breath when measured in liquid; Many VOCs (Volatile Organic Compounds) —some potential biomarkers—are odorless but detected using methods akin to biological olfaction, inspiring the term “electronic nose” (e-nose). As natural smell operates in a moist environment, incorporating a liquid medium offers a biomimetic boost to sensitivity and selectivity. Breath analysis is a non-invasive method for the study of many diseases with the aim of implementing rapid and frequent patient’s diagnosis, prognosis and follow-up (Horváth et al., 2017). This methodology is well-established in the field of the biomedical research (Ahmed et al., 2018; Di Natale et al., 2014; Pennazza and Santonico, 2018). The next step is the use in the clinical practice, which is a matter of sensor technology, standardization and very large patient library. Many of the research teams involved in this challenge, have decided to follow their own technological path in terms of sensor device and collection apparatus, testing their techniques in large clinical studies (Simpson et al., 2019; Broza et al., 2017; Di Gioia et al., 2023; Binson et al., 2021a; Binson et al., 2024a; Daulton et al., 2021). Following the same strategy, the statement to be achieved by this work is to report the first step of a large clinical study covering 5 years and including 863 subjects, with 7 different diseases. The large clinical study responds to the need of a reference library of breath-prints, while the characterization of the exhaled breath sample in liquid responds to the technological need of deeply analyzing the biological specimen with a multi-sensor approach. This approach focuses on two essential elements for successfully translating research into clinical practice: standardized experimental protocols and consistent procedures. These form the foundation of a technological roadmap that must also cross key engineering and regulatory challenges.

## 2 Methods

### 2.1 Instruments

The multi-sensor platform, named BIONOTE (Santonico et al., 2013), is composed of two sensor systems: BIONOTE-L and BIONOTE-V, respectively for the measure of liquids (L) and volatiles (V).

The BIONOTE-L consists of a voltammetric sensor used for liquid analyses (Di Gioia et al., 2023). It comprises a Screen-Printed Electrode probe (SPE; DRP-250B, Metrohm DropSens, Oviedo, Spain) with a working electrode made of gold, a counter electrode made of platinum, and a reference electrode made of silver. This sensing platform is paired with a dedicated electronic interface responsible for supplying the input signal and recording the resulting output data.

When the SPE is immersed in a solution, an input signal is applied, featuring a triangular waveform ranging from +1 V to −1 V. This waveform induces oxidative-reduction (oxi-reduction) phenomena involving the analytes dissolved in the aqueous medium. The system captures the current generated by the electrons participating in these reactions and converts it into voltage using a trans-impedance circuit. The input signal’s frequency is set at 0.01 Hz, and 500 samples are collected per measurement. Each sample undergoes at least five independent analysis cycles. The measurements are performed by an operator using a smartphone running a specific App designed and developed by the authors. The smartphone is wireless connected to the BIONOTE-L via Bluetooth.

The BIONOTE-V is an array of eight Quartz MicroBalance sensors (QMB), functionalized with 8 different anthocyanins (Santonico et al., 2013). The sensor outputs are the features extracted by the eight curves. The main feature is the frequency shift registered between a cleaning starting phase where the chemical interactive material is interacting with an inert gas and no molecules are adsorbed on the material surface and a final stable phase in which the volatiles adsorbed on the chemical interactive material have reached an adsorption-desorption equilibrium with the gas delivered in the sensor cell. The measure protocol for the exhaled breath collection and analysis via BIONOTE-V is reported in the next section.

### 2.2 The collection and measurement methods

**FIGURE 1:**
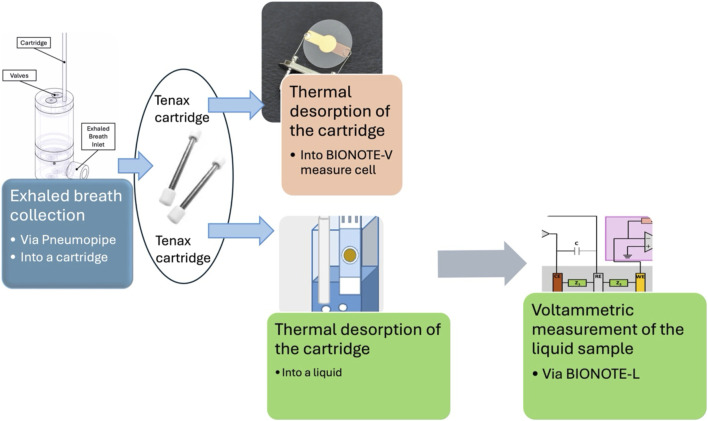
Flow chart reporting the experimental process followed for sampling exhaled breath and measuring it as volatile (VM) and in liquid (LM).

The goal of measuring the same specimen, exhaled breath, with two different methods, asks to design two different collection methods: a method for the analysis of exhaled breath as it is (volatile), that we will name in the following as VM (Volatile Method); a method for the analysis of exhaled breath in liquid matrix, that we will name in the following as LM (Liquid method). These methods have been detailed in previous works (cited here for reference), but for clarity, the flow chart in Figure 1 provides an overview of the set-up for VM and LM.

The method used for the collection and analysis of exhaled breath and its measurement via BIONOTE-V, is the same described by the author in Pennazza et al. (2014).

The method used for the collection and analysis of exhaled breath and its measurement via BIONOTE-L by diluting the collected VOCs into a liquid matrix has been described in Pennazza et al. (2017), and here used with a modification: the transfer of the exhaled breath into the liquid solution was performed via the tenax cartridge. Thus, the individual did not breath directly into the liquid solution (as in Pennazza et al. (2017)), but in a tenax cartridge, in order to standardize the collection procedure with respect to the VM: which is the one running since Pneumopipe introduction and already tested with hundreds of patients in many clinical experiments and which ensures a more stable, reproducible and comfortable collection of exhaled breath. The desorption of the tenax cartridge is performed via the same apparatus used for the BIONOTE-V (Pennazza et al., 2014), but the endpoint is the vial containing the solution and the SPE of the BIONOTE-L, as described in Pennazza et al. (2017). Regarding the reproducibility of this method, as stated in Pennazza et al. (2017), repeated measurements of the same exhaled breath appear to be consistent.

### 2.3 Statistics

**FIGURE 2:**
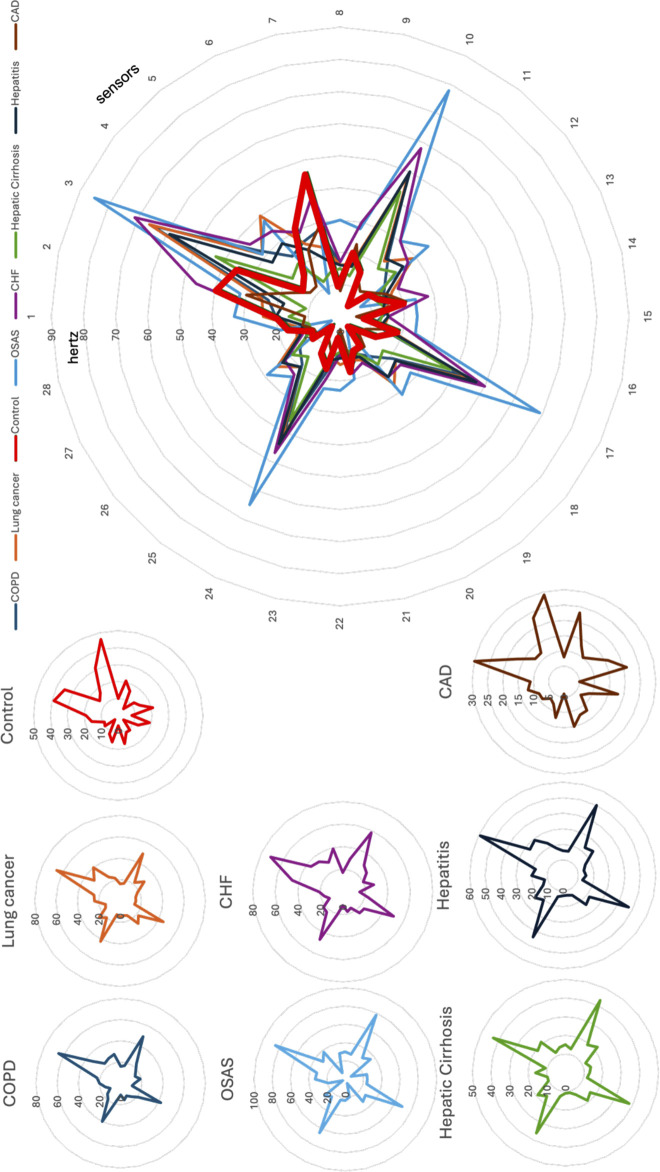
Breathprints of eight individuals (randomly) extracted from the breathprint library: one for each of the eight categories: Controls, COPD, Lung cancer, OSAS, CHF, Hepatic Cirrhosis, Hepatitis, CAD. The reliability of breathprint characteristics, in relation to the diseases (conditions) they represent, is ensured by the demonstrated reproducibility of the method (Incalzi et al., 2012).

The breath fingerprints (from VM and from LM) were represented using a radar-plot (VM), where each radius represents one of the 32 BIONOTE-V sensor responses and its length the magnitude of response in Hertz, and a Voltammogram (LM), where each current value on y-axis is the response of the sensor to the correspondent Voltage input (on the x-axis). Reproducibility of the VM method has been tested in Incalzi et al. (2012), and it can support the representativity of the radar-plots randomly selected to be shown in Figure 2 (in the first part of the results section).

The data set of the VM study is very large. As anticipated in the title, here an integrative analysis of 5 years of exhaled breath–based diagnostics for seven diseases is presented.

We used a discrimination process with three distinct analysis steps to analyze and elaborate the data. Each step is based on multiple K-Nearest Neighbor (KNN) models for classification. The first two steps deal with a binary classification task, while the last step has the goal of a multiclass discrimination problem.

To evaluate the system’s performance, we employed 5-fold cross-validation. This technique involves dividing the data into five subsets. The model is trained on four subsets and tested on the remaining subset. This process is repeated five times, each time using a different subset for testing. This approach ensures that the model is evaluated on unseen data. The classification task of step 1 is obviously unbalanced, and this is due to the choice of using all the data relative to the different diseases vs. control individuals.

A subgroup of the patients of the VM study were enrolled also for the LM study. The number is too low to allow advanced techniques, thus Partial Least Square Discriminant Analysis (PLS-DA) was used.

### 2.4 Study population

The population here considered pertains to a large multi-clinical study covering 5 years and including 863 subjects in two different centers. The subjects enrolled can be divided in 8-subgroups, as reported in Table 1. These groups are referred to five different studies already published. The study protocols adhered to the principles outlined in the Declaration of Helsinki, and they were approved by the Campus Bio-Medico Ethical Committee (Protocol No. 29/18 OSS ComEt CBM; protocol number: 30/15 PAR CMB; 4711 CBM; 21.16 TS ComEt CBM), and all patients provided written informed consent.

**TABLE 1:** List of the 863 subjects enrolled for the clinical study: 38 of them are control individuals, the other 825 are affected by 6 different diseases.

Typology	Number of subjects	Published study
Control	38	All the following
COPD (Chronic obstructive pulmonary disease)	281	Scarlata et al. (2018), Finamore et al. (2018b)
Lung Cancer	186	Rocco et al. (2016)
OSAS (Obstructive sleep apnea syndrome)	134	Scarlata et al. (2017)
CHF (Congestive heart failure)	78	Finamore et al. (2018a)
Hepatic cirrhosis	70	De Vincentis et al. (2017), De Vincentis et al. (2019)
Hepatitis	34	De Vincentis et al. (2017), De Vincentis et al. (2019)
CAD (Coronary artery disease)	42	Segreti et al. (2020)

In the lung cancer experiment, 15 control individuals and 15 patients affected by lung cancer have been double sampled in order to analyze the exhaled breath also in liquid, by BIONOTE-L. In a previous work Pennazza et al. (2017), the method was validated with control individual. Here the method was applied to a basic discrimination step: lung cancer affected individuals vs. control individuals. Considering that, as described in the methods section, the transfer of the exhaled breath into the liquid solution was performed via the tenax cartridge, this procedure was realized for a sub-group of the lung cancer experiment population. The reason is exclusively due to chronological aspects of the different clinical experiment with respect to the development of the LM.

## 3 Results

### 3.1 Volatile test

The dimensionality of the collected data asks for a multivariate data analysis technique, as described in the methods, but the picture given by the radarplot representing the fingerprints of the different exhaled breath gives a suggestive idea of the differences supporting possible discriminations. As example, Figure 2 illustrates the superposition of 7 exhaled breath fingerprints (breathprints), each one of them randomly extracted by the library of 863 breathprints, one for each of the eight conditions here represented: controls, COPD, Lung cancer, OSAS, CHF, Hepatic Cirrhosis, Hepatitis CAD; each of them is also represented individually in Figure 2.

The breathprint of the control individual is evidenced with a bold red line, so that it is evident the difference with the other seven breathprints relative to seven patients affected by seven diseases.

**FIGURE 3:**
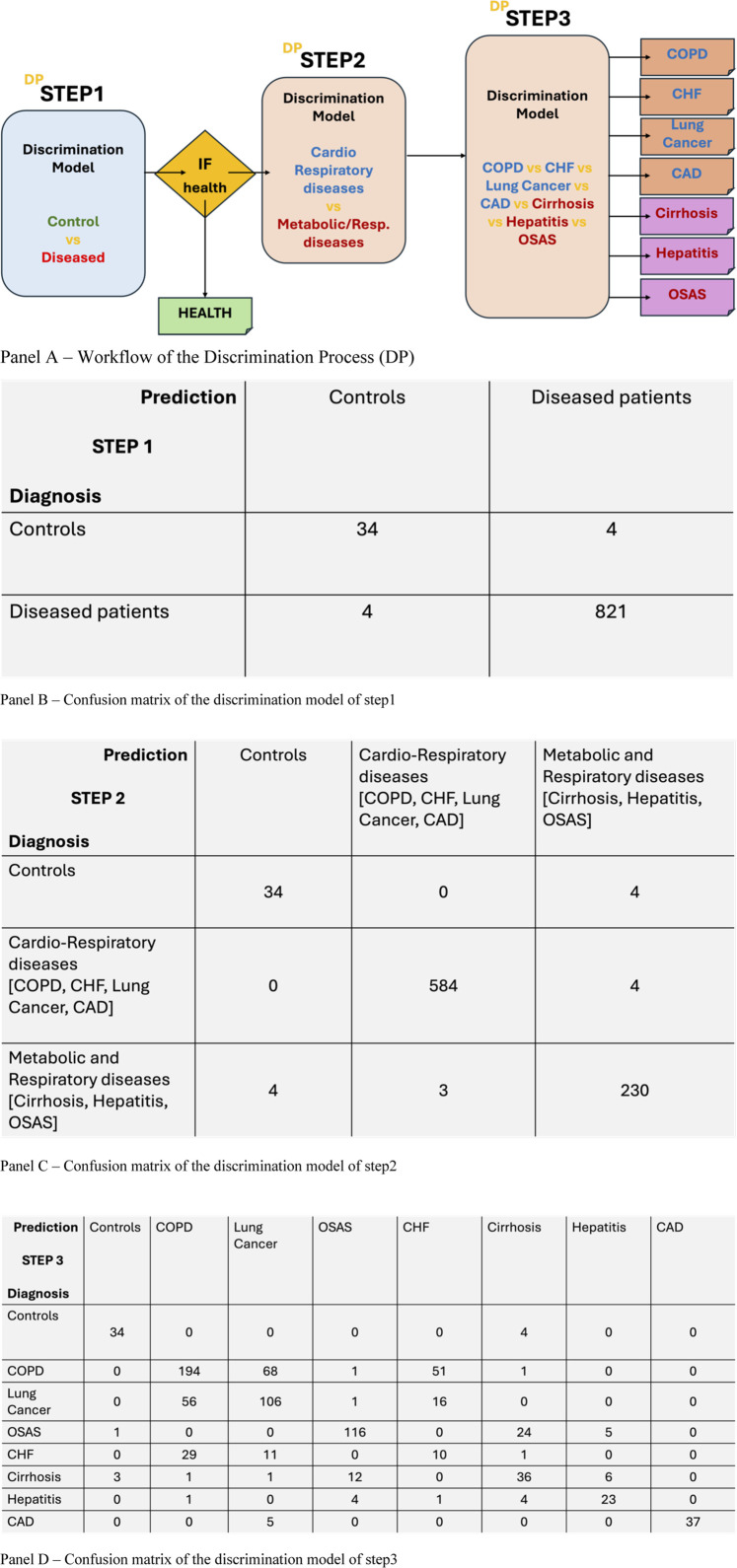
**(A)** workflow of the Discrimination Process (DP) with the three steps of elaboration/discrimination: Step 1: control vs. disease; Step 2: cardio-respiratory disease vs. metabolic-respiratory diseases; Step 3: discrimination of the specific health-condition; **(B)** Confusion matrix of the control/disease discrimination; **(C)** Confusion matrix of the disease typology (cardio-respiratory/metabolic-respiratory) discrimination; **(D)** Confusion matrix of 8 specific health conditions discrimination.

A discrimination process (DP) composed of three independent KNN-based classification steps was employed for data analysis. The first two steps addressed binary classification tasks, while the third handled a multiclass task. To assess the system’s performance, a 5-fold cross-validation was conducted. This technique involves dividing the dataset into five folds, training the system on four folds, and evaluating it on the remaining fold. The process workflow is reported in Figure 3, panel A. This process is repeated five times, ensuring that each fold is used for testing exactly once. The results of this validation are presented in Figure 3, panels B, C and D.

Panel 3B, the confusion matrix of step 1, shows a key-point of the work. It says that, with a significant number of individual (863) including a 5% of control individuals, and seven different diseased conditions, with percentages ranging from 4% to 38% of the total population, the breathprint of a diseased patient, independently on the specific disease, has a systemic peculiarity which makes the BIONOTE-V able to correctly discriminate it with respect to a healthy condition, with the following performances: sensitivity 99%; specificity 99%; NPV 99%; PPV 99%; Accuracy 99%.

Panel 3C, the confusion matrix of step 2, shows that the breathprint of a diseased patient, even presenting a global profile which is different by a control individual, contains characteristics which are distinctive of disease typology. Specifically, four of the diseases represented in the total population, are referred to cardio-respiratory diseases, accounting for about 68% of the total population: COPD, CHF, Lung Cancer, CAD; three of the diseases represented in the total population, are referred to metabolic-respiratory diseases, accounting for about 27% of the total population: OSAS, Cirrhosis, Hepatitis. It is significant that the percentage of correct classification for controls is the same as step 1. The percentage of correct classification for cardio-respiratory diseases is of 93%. The percentage of correct classification of metabolic-respiratory diseases is of 84%. The total accuracy of the discrimination process performed at step 2 is of 98%.

Panel 2D, the confusion matrix of step 3, shows that the breathprint of a diseased patient, even presenting a global profile which is different by a control individual, contains characteristics which are distinctive of each specific disease, among the ones selected in this study. Specifically, COPD, CHF, Lung Cancer, OSAS, Cirrhosis, Hepatitis. It is significant that the percentage of correct classification for controls is the same as step 1 and 2. The percentage of correct classification for each disease is: 84% for COPD, not significant for CHF, 59% for Lung Cancer, 79% for OSAS, 61% for Cirrhosis, 70% for Hepatitis, 88% for CAD. The total accuracy of step 3 discrimination process is of 64%. It is worth to remark the various cases of misclassification among diseases. COPD’s highest number of misclassified are registered with Lung Cancer. The same happens with the lung cancers, whose misclassified mainly occurs with respect to COPD. CHF is the only disease which is not correctly classified: the most frequent misclassification for CHF is COPD, which is, indeed, a frequent comorbidity for CHF patients. Cirrhosis misclassification is often in favor of OSAS. Hepatitis misclassifications are not polarized by any other disease in particular, with low number of misclassified distributed among all the other diseases. CAD is only confused in the 12% of the cases with lung cancer. In the discussion each result is compared with each single previous study conducted with the same technology (reported in Table 1 in the methods section).

### 3.2 Liquid test

The results obtained by the elaboration of the 30 individuals enrolled as population for the pilot test of exhaled breath analysis in liquid has given very promising scores, even if based on very low numbers. Besides, it is worth remarking that, to our best knowledge, this is the first experiment applying this methodology on a real sub-population of controls and lung cancer patients participating to a larger clinical study on exhaled breath (see previous section). Authors already published a methodological study on exhaled breath in liquid (Pennazza et al., 2017), and this is the first application of the method in a clinical context. The performance obtained are comparable with the current breathprint analysis by BIONOTE-V. From Table 2, it can be calculated that Sensitivity and specificity are about 87% and 100% respectively; PPV is of 100% and NPV is of 88%.

**TABLE 2:** Confusion matrix of the pilot test of the measurements of exhaled breath in liquid.

Reference	Prediction		Model performance
	Controls	Lung cancer patients	
Controls	15	0	PPV: 100%
Lung cancer patients	2	13	NPV: 88%
​	Sensitivity: 87%	Specificity: 100%	Accuracy: 93%

**FIGURE 4:**
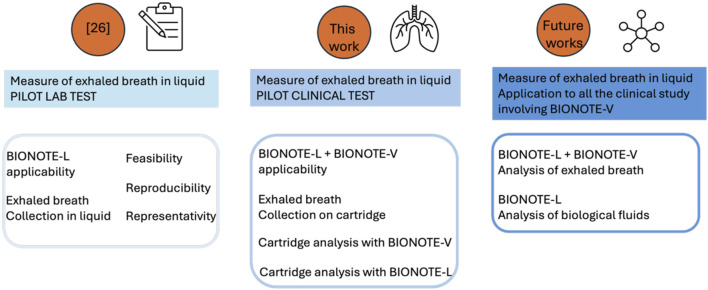
Workflow of the running process to couple BIONOTE-L and BIONOTE-V in the breathprinting of exhaled breath.

The significance of this result lies not in the scale of the experiment, but rather in its feasibility. We’ve demonstrated a dual-technology measurement approach, applicable to future studies. The goal of a multi-sensor strategy is twofold: measuring multiple biofluids with specialized sensors and measuring the same biofluid with diverse sensors. While the former has been explored in previous work Pipita et al. (2020), Schirinzi et al. (2021), Lelli et al. (2018), Di Gioia et al. (2023), the latter, initiated in Pennazza et al. (2017), is further developed here and depicted in Figure 4.

## 4 Discussion

The results of this work show that the multi-sensor platform consisting of BIONOTE-L and BIONOTE-V, supported by a suitable and standardized sampling procedure for exhaled breath and a reproducible measuring protocol to analyze exhaled breath as it is (volatile) and in a liquid matrix, can be effectively used in a multi-center study for discriminating many different diseases with an unobtrusive strategy. This conclusion, which represents a starting point for a necessary clinical study to assess feasibility and reliability in daily medical practice, is provided as a sum of steps reached by this work: BIONOTE-V effectiveness with respect to more than one disease (multi-disease context). The applicability of an unobtrusive standard procedure for exhaled breath collection, storage and shipment in the frame of a multi-center experiment, presenting different diseased conditions (meaning different frailty occurrences, different operative difficulties and different surrounding environments). The possibility, and opportunity, to extend sensor technology to a multi-sensor empowerment including another sensor working principle suitable for many other biological fluids but never used so far for exhaled breath. In order to support these conclusions, it is useful to compare the discrimination ability here obtained with the performances evidenced in each one of the previous study (see Table 1) whose population has contributed to built-up the breathprint library here used. With respect to studies on COPD and CHF (Finamore et al., 2018a; Scarlata et al., 2018; Finamore et al., 2018b), the results confirm, and even strengthen, the previously found accuracy of 81% (95% CI 69%–89%) in discriminating CHF from Controls. Indeed, on a larger sample, no case of CHF is misclassified as a control, nor is any control misclassified as CHF. Conversely, while previous studies showed a significant, even low, discriminative ability between COPD and CHF (i.e. 69% of accuracy, 95% CI 57%–80%), in this study the vast majority of CHF patients is misclassified as having COPD or, less frequently, lung cancer. This discrepancy might be partially explained by diagnosed and misdiagnosed comorbidities; indeed, CHF and COPD frequently coexist, with a prevalence of heart failure in COPD ranging between 20% and 30% (Mannino et al., 2024) and a prevalence of COPD in heart failure of 10%–40% (Mooney et al., 2021), in line with the findings. Likewise, lung cancer patients are often misclassified as COPD, a common comorbidity, often reported by enrolled patients (Rocco et al., 2016), due to shared risk factors like cigarette smoking. Similar results can be found in literature also in works by other research groups studying COPD/lung cancer mixed (Binson et al., 2021b) and lung cancer (Binson et al., 2024b) populations.

With respect to the OSAS study (Scarlata et al., 2017), results are in line with the high discriminative accuracy between OSAS and Controls, which was around 100%, but contrast with the low discriminative accuracy found with COPD. Indeed, on a larger sample, OSAS is usually misclassified with hepatic diseases rather than COPD, which might be explained by the metabolic effect of OSAS.

In the field of hepatology (De Vincent et al., 2017; De Vincentis et al., 2019), BIONOTE-V was tested in two sequential studies aimed at verifying its potential diagnostic and prognostic abilities. Indeed, distinctive breath prints were shown to characterize patients with liver cirrhosis (LC), non-cirrhotic chronic liver disease (NC-CLD) and healthy controls (sensitivity 86.2% and specificity 98.2% for CLD vs. healthy controls, and 87.5% and 69.2% for LC vs. NC-CLD), and, among LC patients, the different functional classes as described by the Child-Pugh score (De Vincentis et al., 2019). Subsequently, LC patients were followed-up for nearly 2 years and clinically relevant events of hepatic decompensation or death were collected. Selected breath-print clusters were disclosed to be associated with a significantly increased risk of mortality and hospitalization, even in multiple adjusted models (adjusted [a]HR 2.8, 95% CI 1.1-7.0 for mortality and aHR (adjusted Hazard Ratio) 2.2, 95% CI 1.1-4.2 for hospitalization) (De Vincent et al., 2017). Overall, these studies highlight the potential use of breath analysis for the fast detection of liver disease, for staging its severity and tracking health trajectories during time (De Vincentis et al., 2019).

With respect to CAD (Segreti et al., 2020) A cross-sectional study evaluated the diagnostic potential of VOC analysis using the BIONOTE-V device in the context of chronic coronary syndromes (CCS). The study involved 42 patients undergoing invasive coronary angiography (ICA). Based on ICA results, the patients were divided into two groups: those requiring myocardial revascularization and those not requiring it.

BIONOTE-V successfully identified 18 out of 23 patients requiring myocardial revascularization (sensitivity: 78.3%) and 13 out of 19 patients not requiring revascularization (specificity: 68.4%). The breath analysis findings demonstrated a strong correlation with the complexity of CAD, as evaluated by SYNTAX scores.

Moreover, patients correctly classified by BIONOTE-V exhibited elevated platelet activation markers, specifically platelet distribution width and mean platelet volume, highlighting its ability to detect severe coronary conditions and associated platelet activity.

These findings suggest that CCS patients have a distinctive exhaled breath profile that can be effectively detected by BIONOTE-V. This technology offers a rapid, cost-effective, and non-invasive diagnostic method with the potential to identify the different clinical presentations of CCS.

Despite the limited sample size, the BIONOTE-L has yielded promising results. We’ve adapted the liquid breath analysis protocol from Binson et al. (2021a) to enable a unique collection method and dual measurements (BIONOTE-V and -L). The results align with previous lung cancer studies (Rocco et al., 2016). Although the sample size is limited, the representativeness of single-collection, dual-tested samples is noteworthy and positions this approach for broader clinical application in future investigations.

The reduction of performance can be explained by the significantly increased complexity of discriminating among eight distinct health conditions in Step 3 compared to the simpler binary classifications in earlier steps. This complexity leads to an accumulation of errors in the hierarchical classification process and a greater likelihood of overlapping features between the more granular subclasses. Furthermore, many of the diseases share underlying biological mechanisms, risk factors, or are common comorbidities, resulting in similar volatile organic compound (VOC) profiles that challenge distinct classification. Finally, the inherent heterogeneity within each disease category, encompassing variations in severity and patient responses, creates less defined clusters, making precise decision boundaries for each specific illness more difficult to establish. A supervised analysis technique as the ones based on artificial intelligence, should be used to make a step beyond the biological mechanism association to the breathprint, by tracking specific characteristics (Liu et al., 2025; Wasilewski et al., 2024) and parameters variations to the modification registered in the multidimensional data given by the BIONOTE.

A final remark must be devoted to a term cited in the title and currently strategic: sustainable. Sustainability in health context could be somewhat misleading and a different approach is needed with respect to other contexts: there is an unavoidable complex trade-off between effectiveness of care, safety, costs, and pollution which opens sustainability spectra in its environmental, social and economic components (Or and Seppänen, 2024). The healthcare system is indeed a major contributor to climate change; healthcare accounts for an estimated 5% of all greenhouse gas emissions worldwide (Rocco, 2024; Pinho-Gomes et al., 2022). Given the complexity of healthcare sustainability, clinicians and researchers must prioritize the development of eco-friendly and clinically effective products and processes. A crucial step is to incorporate carbon footprint measurement and minimization as essential outcomes in randomized clinical trials. This proposed technology, while still in its early stages of development, could serve as a foundational approach towards achieving this goal.

Considering that non-invasive procedures support a remote and frequent monitoring which allowing preventive strategies and at-home follow-up, reduce hospitalization and, obviously, decrease costs and pollution, sustainability can be also evaluated by a Life Cycle Assessment (LCA), which is here roughly applied to give a first hint for successive elaborations. Using thesoftaware SimaPro the carbon footprint for BIONOTE fabrications has been calculated (it is reported in Table 3). Also BIONOTE and HPLC (high-performance liquid chromatography) 1-h utilization footprints have been calculated and compared (see Table 4).

**TABLE 3:** LCA assessment of BIONOTE fabrication in terms of carbon footprint [Kg CO_2 eq_].

Impact category	Unit	Total	Ceramic support	Electronic component, passive	Electronic component, active	Gold	Silver	Pt	Electricity, low voltage
Carbon footprint	% CO_2_ eq	100%	1,66%	1,81%	72,04%	21,94%	0,74%	3,94%	0,06%

**TABLE 4:** LCA assessment of BIONOTE and HPLC 1 h utilization in terms of carbon footprint [Kg CO_2 eq_].

Unit	BIONOTE-L	HPLC
kg CO_2_ eq	0,00116	0,307

The choice of HPLC is given by the fact that this is the laboratory instrument routinely used in the clinical context which is most like BIONOTE (Rodríguez-Aguilar et al., 2019). From this analysis the utilization of BIONOTE in a clinical context should reduce of two order of magnitude Carbon footprint in terms Kg CO_2 eq_.

## Data Availability

The raw data supporting the conclusions of this article will be made available by the authors, without undue reservation.
